# Mobile, alltagsnahe digitale Technologien für die Prävention der Alzheimer-Demenz: kognitive Gesundheit und kognitive Sicherheit

**DOI:** 10.1007/s00115-023-01478-4

**Published:** 2023-04-28

**Authors:** Emrah Düzel, Jochen René Thyrian

**Affiliations:** 1 Institut für Kognitive Neurologie und Demenzforschung, Medizinische Fakultät, Universität Magdeburg, Leipziger Str 44, 39120 Magdeburg, Deutschland; 2 Deutsches Zentrum für Neurodegenerative Erkrankungen, Standort Greifswald, Greifswald, Deutschland; 3 Lebenswissenschaftliche Fakultät (LWF), Universität Siegen, Siegen, Deutschland

**Keywords:** Präventive Maßnahmen, Risikoreduktion, Versorgungskonzepte, Medizinische Interdisziplinarität, Gesundheitskompetenz, Preventive measures, Risk reduction, Treatment concepts, Interdisciplinary medicine, Health competence

## Abstract

Es ist allgemein akzeptiert, dass die Versorgung der Alzheimer-Erkrankung durch präventive Maßnahmen zur Risikoreduktion flankiert werden sollte, um kognitive Funktionen möglichst lange aufrecht zu erhalten. Aber sowohl die Forschung als auch die Entwicklung von Versorgungskonzepten stehen hier vor Herausforderungen. Zum einen erfordert die präventive Risikoreduktion ein hohes Maß an medizinischer Interdisziplinarität der Neurologie und Psychiatrie mit anderen Disziplinen, zum anderen müssen Patienten ein hohes Maß an Gesundheitskompetenz entwickeln sowie Eigenmotivation und Adhärenz aufbringen. In diesem Konzeptpapier geht es um die Frage, wie mobile, alltagsnahe digitale Technologien helfen können, diese Herausforderungen zu adressieren. Die zentrale Prämisse ist die über Disziplinen hinweg koordinierte Strukturierung der Prävention mit den Schwerpunkten *kognitive Gesundheit *und *kognitive Sicherheit*. Kognitive Gesundheit fokussiert auf eine Reduktion lebensstilassoziierter Risikofaktoren. Kognitive Sicherheit betrifft die Minimierung iatrogen verursachter Nebenwirkungen auf kognitive Funktionen. Bei den digitalen Technologien, die in diesem Zusammenhang relevant werden, handelt es sich um mobile Smartphone- oder Tablet-basierte Apps zur alltagsnahen und hochfrequenten Erfassung kognitiver Funktionen, Apps, die als Companion-Technologien die Implementierung von Lifestyleänderungen coachen können, Apps, die bei der Reduktion iatrogener Risiken assistieren können und solche, die Gesundheitskompetenz von Patienten und Angehörigen verbessern können. Entsprechende Medizinprodukte sind in ihrem Entwicklungsstand unterschiedlich weit fortgeschritten. Daher geht es in diesem Konzeptpapier nicht um eine Produktübersicht, sondern um das prinzipielle Zusammenspiel potenzieller Lösungen in der Prävention der Alzheimer-Demenz in den Bereichen kognitive Gesundheit und kognitive Sicherheit.

## Abstract (en)

It is generally accepted that the treatment of Alzheimer’s disease should be flanked by preventive measures for risk reduction in order to maintain cognitive functions for as long as possible; however, the research and development of treatment concepts are both faced with challenges. The preventive risk reduction necessitates a high level of coordination of neurology and psychiatry with other disciplines. Also, patients must develop a high level of health competence and summon up self-motivation and adherence. This concept article deals with the question of how mobile everyday-life digital technologies can help to address these challenges. The core prerequisite is the interdisciplinary coordinated structuring of prevention with the focus on cognitive health and cognitive safety. Cognitive health relates to a reduction of risk factors associated with lifestyle. Cognitive safety concerns the avoidance of iatrogenic side effects on cognitive functions. Digital technologies that are relevant in this context are mobile apps based on smartphones or tablets for everyday-life and high-frequency recording of cognitive functions, apps that can coach the implementation of lifestyle changes as companion technologies, apps that can assist in the reduction of iatrogenic risks and those that can improve the health competence of patients and relatives. The state of development of such medical products is at different stages of progress. Therefore, this concept article does not provide a review of existing products but rather deals with the fundamental interplay of potential solutions in the prevention of Alzheimer dementia in the areas of cognitive health and cognitive safety.

## Hintergrund

Kognitive Beeinträchtigungen im Rahmen der Alzheimer-Erkrankung können durch den Verlust von Neuronen und Neuropil und als Folge gestörter Netzwerkfunktion im Gehirn auftreten [5]. Ursächlich sind hier neben der Alzheimer-assoziierten Tau- und Amyloid-Pathologie auch Störungen der Proteostase, Störung der Gliafunktion, Verlust oder Überfunktion von Neurotransmittern, mitochondriale Dysfunktion, Inflammation, vaskuläre Dysfunktion und Störung der Clearance [18]. In der Auslösung und Aufrechterhaltung dieser pathologischen Prozesse können extrazerebrale Faktoren eine wichtige Rolle spielen [25]. Dazu gehören Erkrankungen des Herz-Kreislauf-Systems, der Nieren, der Leber und anderer Organe sowie iatrogene Maßnahmen wie Narkosen, Sedierungen, Immobilisierung und Medikamentennebenwirkungen. Schließlich spielen lebensstilassoziierte Risiken, wie Bewegungsmangel, ungünstige Ernährung, soziale und kognitive Deprivation eine modifizierende Rolle.

**Figure 1:**
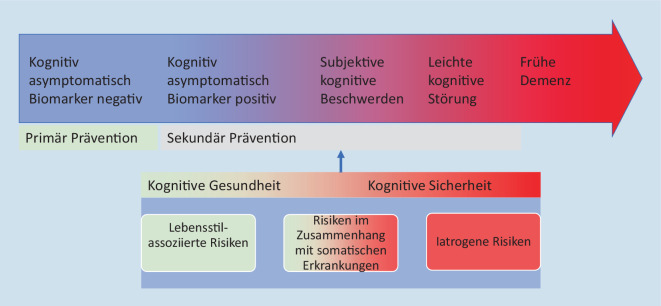

Aus dieser pathophysiologischen Komplexität ergibt sich, dass präventive Maßnahmen multiple Dimensionen adressieren sollten (Abb. 1). Eine Dimension ist die Verbesserung kognitiver Funktionen durch eine Anpassung des Lebensstils, Reduktion lebensstilassoziierter Risikofaktoren und somit Optimierung der körperlichen Gesundheit. Diese Dimension betriff also die *kognitive Gesundheit*. Eine andere Dimension ist die Verhinderung iatrogen verursachter Nebenwirkungen auf kognitive Funktionen. Diese Dimension betrifft vor allem die *kognitive Sicherheit *von Patienten. Risiken durch somatische Erkrankungen können sowohl die kognitive Gesundheit als auch die kognitive Sicherheit betreffen. Im Folgenden wird betrachtet, wie digitale Technologien helfen können, die Prävention der Alzheimer-Erkrankung in beiden Dimensionen, kognitive Gesundheit und kognitive Sicherheit, zu verbessern.

Smartphone-basierte App-Technologien können das Assessment und Monitoring kognitiver Defizite erleichtern [33] und sie können lebensstilbasierte Interventionen coachen (z. B. [20]). Darüber hinaus ist auch plausibel, dass sie geeignet sind, Risikofaktoren zu tracken und zu analysieren, wie dies z. B. bei kardiovaskulären Erkrankungen schon verfügbar ist (Beispiel HerzFit-App der Deutschen Herzstiftung). Schließlich können App-basierte Companion-Technologien die Demenzprävention für Patient:innen und Angehörige unterstützen [8]. Vielfach befinden sich diese Technologien jedoch noch im Entwicklungsstadium und sind vor allem im Hinblick auf Prävention bezüglich ihrer Versorgungseffekte und Auswirkungen auf kognitive Trajektorien nicht validiert.

## Strukturierung relevanter Risikofaktoren

Für die Betrachtung, wie digitale Technologien in der Prävention der Alzheimer-Demenz unterstützen können, ist es hilfreich, relevante Risikofaktoren entsprechend möglicher Implementierungsszenarien zu gliedern:

1. lebensstilassoziierte Risikofaktoren und *kognitive Gesundheit,*
2. Risiken im Zusammenhang mit somatischen Erkrankungen und *kognitive Gesundheit und Sicherheit,*
3. iatrogene Risiken und *kognitive Sicherheit.*

Diese drei Bereiche erfordern unterschiedliche Implementierungsszenarien und Technologien. Lebensstilbasierte Interventionen sollten personalisierbar sein und ein vornehmlich patientenzentriertes Design haben. Das Erfassen und Modifizieren von Risiken im Zusammenhang mit somatischen Erkrankungen erfordert eine enge Anbindung an die elektronische Patientenakte und Interaktion mit medizinischem Fachpersonal. Bei iatrogenen Risiken und Patientensicherheit müssen sowohl Patienten als auch medizinisches Fachpersonal angesprochen werden.

Iatrogene Risiken erfordern ein zeitlich engmaschiges Monitoring

Alle drei Bereiche erfordern zudem unterschiedliche Arten des kognitiven Monitorings. Digitale Technologien zur Umsetzung lebensstilassoziierter Interventionen z. B. erfordern kein engmaschiges (z. B. wöchentliches) kognitives Monitoring, da nicht zu erwarten ist, dass derartige Interventionen zu einer kurzfristig messbaren kognitiven Veränderung führen. Stattdessen sind diese Interventionen langfristig ausgelegt (ca. 6–12 Monate) und das kognitive Monitoring kann mit entsprechendem zeitlichem Abstand erfolgen. Im Gegensatz dazu erfordern iatrogene Risiken, wie z. B. die postoperative kognitive Dysfunktion (POCD), ein zeitlich engmaschiges Monitoring, um die individuelle Auswirkung auf die Kognition zu erfassen, eine potenzielle Erholung oder gar Akzeleration der Alzheimer-Progredienz zu erfassen und diese Daten bei zukünftigen Therapieentscheidungen zu berücksichtigen.

## Digitale Erfassung der Kognition

Die mobile, alltagsnahe und nichtsupervidierte Selbstmessung der Kognition per Smartphone wird derzeit intensiv entwickelt und erforscht [33, 41]. Weltweit sind einige Lösungen bereits als Medizinprodukte in Entwicklung und in Deutschland ist z. B. die neotivCare App zugelassen, um eine für eine leichte kognitive Störung typische Gedächtnisstörung zu erfassen [2, 3].

Mithilfe der digitalen Messmethode per Smartphone wird die Diagnostik und Verlaufsmessung kognitiver Störungen in die häusliche Umgebung überführt und von zeitlichen und terminlichen Einschränkungen befreit [3, 14]. Das heißt, die Testung wird aus der Arztpraxis oder dem Krankenhaus verlagert in die gewohnte, alltägliche Umgebung der Betroffenen und ist für einen längeren Zeitraum verfügbar. So kann die Technologie ein wesentlich repräsentativeres Bild der Kognition abbilden, indem eine Momentaufnahme durch einen Diagnosezeitraum von bis zu mehreren Wochen abgelöst wird und die Testung alltagsnah erfolgt. Der lange Diagnosezeitraum verbessert nicht nur die Repräsentativität und Verlässlichkeit der Daten, sondern auch die Nutzbarkeit für Patienten. Der lange Zeitraum ermöglicht zudem eine Redundanz, wodurch jeder einzelne Test weniger ins Gewicht fällt. Das bedeutet, dass gerade in der Anfangsphase der Testungen die Patienten sich keine Sorgen machen müssen, dass technische Probleme oder Verständnisprobleme ihre Ergebnisse negativ beeinflussen. Sie müssen also die Nutzung der App nicht abbrechen, sollten die ersten Tests nicht gut gelingen. Gleichzeitig stellt diese Aussicht auf eine stabile, fluktuationsbereinigte Erfassbarkeit der Kognition und ihrer Veränderung eine potenziell wichtige Motivation für Arzt und Patient dar, Prävention zu betreiben und ihre Effekte auf die Kognition zu monitorieren.

### Indikationsstellung

Die Alzheimer-Erkrankung ist definiert durch eine positive Amyloid- und Tau-Pathologie und hat einen prädemenziellen Verlauf von bis zu 20 Jahren. In dieser Zeit kann die klinische Progression in drei Stadien (Abb. 1) untergliedert werden [23]:

- Im Stadium 1 sind Betroffene asymptomatisch und kognitiv unauffällig.
- Im Stadium 2 bestehen subjektive Beschwerden über eine nachlassende Kognition, wie z. B. die Wahrnehmung einer zunehmenden Gedächtnisschwäche, aber die kognitive Leistungsfähigkeit liegt neuropsychologisch noch im Normbereich.
- Im Stadium 3 liegt die kognitive Leistungsfähigkeit in einer Domäne, meist in der Gedächtnisleistung, unterhalb der Altersnorm, aber die Betroffenen sind noch in der Lage ihren Alltag unabhängig zu bewältigen [23].

Maßnahmen zur Prävention der Alzheimer-Erkrankung in den klinischen Stadien 1 bis 3 bezeichnet man als Sekundärprävention (Abb. 1) einer Demenz [17].

Prävention und krankheitsmodifizierende Therapien bedürfen einer frühen Diagnose

Die leichte kognitive Störung (Abb. 1) ist ein wichtiges klinisches Stadium sowohl für Präventionsmaßnahmen, die eine kognitive Verschlechterung verlangsamen können und heute schon umsetzbar sind, als auch für krankheitsmodifizierende Therapien, z. B. mit Amyloid-Antikörpern. Letztere stehen möglichweise zeitnah zur Verfügung [12]. Daher ist es aus medizinischer Sicht wichtig, die Diagnostizierbarkeit der leichten kognitiven Störung in der Versorgung zu verbessern, zu vereinfachen und durch digitale Technologien in der Breite verfügbar zu machen. Dadurch kann die Versorgung in Fachkliniken entlastet, die Zuweisung von Patienten mit einer leichten kognitiven Störung an Spezialisten verbessert und Wartezeiten verringert werden. Die Verfügbarkeit dieser Diagnosemöglichkeit in der Breite der Versorgung könnte ein wesentlicher Motivator sein, Prävention zu verschreiben und auch die Bereitschaft von Krankenkassen erhöhen, hier zu erstatten. In diesem Zusammenhang werden auch ethische Aspekte einer Frühdiagnose der leichten kognitiven Störung relevant [1].

### Kognitives Baseline-Assessment und Monitoring

Das kognitive Leistungsniveau vor Beginn von Präventionsmaßnahmen und der Verlauf der Kognition sind potenziell wichtige Parameter für die Indikationsstellung, die Auswahl geeigneter Präventionsmaßnahmen und für Entscheidungen darüber, ob/welche Präventionen fortgesetzt oder aufgrund unzureichender Wirkung gestoppt werden sollten. Zum einen ist das kognitive Ausgangsniveau ein wichtiger Risikofaktor für die Vulnerabilität gegenüber somatischen und iatrogenen Risiken. So ist ein wichtiger Risikofaktor für eine POCD oder ein postoperatives Delir das Vorhandensein einer präoperativen kognitiven Störung, wie eine leichte kognitive Störung oder eine Demenz [9, 15]. Es ist daher von Bedeutung, in der Versorgung zu etablieren, ob ein Patient von einer leichten kognitiven Störung betroffen ist. Zum anderen, kann die Beratung darüber, wie intensiv Prävention betrieben wird, davon abhängen, wie progredient der Verlauf der kognitiven Verschlechterung ist.

### Verfügbarkeit im Versorgungspfad

Schließlich erfordert die Multidisziplinarität der Alzheimer-Prävention, dass kognitive Leistungsfähigkeit an verschiedenen Punkten der Versorgung von klinischer Relevanz ist und eine valide, standardisierte Diagnostik derselben verfügbar sein sollte. Es ist wichtig, dass unterschiedlich geschulte Fachkräfte in verschiedenen Settings Zugriff auf ein gemeinsames, standardisiertes Assessment haben, um die eventuell eigenen, individuell angewandten Testinstrumente und klinischen Beobachtungen in Beziehung zu setzen und Vergleiche zu ermöglichen. Hier wird durch die patientenzentrierte und lebensnahe digitale Selbsttestung eine Konstanz erzeugt, die die Vergleichbarkeit kognitiver Leistungsfähigkeit im Verlauf und an unterschiedlichen Stellen des Versorgungspfades wesentlich verbessern kann.

## Lebensstil, somatische Erkrankungen, iatrogene Risiken

Alle drei Aspekte – Lebensstil, somatische Erkrankungen, iatrogene Risiken – spielen in der Sekundärprävention eine wichtige Rolle und greifen ineinander über. Gleichzeitig müssen für alle drei unterschiedliche Akteure eingebunden werden und die digitalen Lösungen müssen sich entsprechend unterscheiden.

### Lebensstilassoziierte Risikofaktoren

Die Hauptadressaten für Präventionen, die auf lebensstilassoziierte Risiken im Kontext der Alzheimer-Erkrankung abzielen, sind Patienten, Angehörige, Hausärzte und behandelnde Fachärzte (meist Neurologie/Psychiatrie). Notwendig sind hier digitale Lösungen, mit denen Verhaltensänderungen induziert, motiviert und begleitet werden.

*Bluthochdruck, Übergewicht, Rauchen, Diabetes *zählen zu modifizierbaren Risikofaktoren, die altersabhängig signifikant mit einem erhöhten Alzheimer-Risiko assoziiert sind [16, 29]. *Bewegungsmangel *(also weniger als 0,5 h von schnellem Gehen, Jogging oder Laufen pro Woche; [24]) ist ebenfalls ein wichtiger Risikofaktor. *Ernährungsbedingte Risiken *beziehen sich auf die Risiken eines westlichen Ernährungsstils und die positiven Wirkungen vitamin- und polyphenolreicher Diäten mit geringer Aufnahme an gesättigten Fettsäuren, wie z. B. eine mediterrane Diät. Auch pflanzenbasierte Diäten wie MIND (Mediterranean-DASH Diet Intervention for Neurodegenerative Delay) und DASH (Dietary Approach to Stop Hypertension) können insbesondere bei Personen mit einem erhöhten Risiko für Herz-Kreislauf-Erkrankungen kognitiv protektiv sein [10]. *Soziale Isolation und kognitive Deprivation *sind ebenfalls lebensstilassoziierte Risikofaktoren, da Personen mit einer längeren Teilhabe am Arbeits- und Sozialleben bessere kognitive Funktionen gegenüber Personen mit einem weniger sozialaktiven Lebensstil zeigen [7]. Personen ohne Hörbeeinträchtigung zeigen eine bessere Aufrechterhaltung der kognitiven Fähigkeiten.

*Kognitives Training *kann sich positiv auf die Performanz in Intelligenztests auswirken [22, 38]. Vor allem in Bezug auf die kognitive Gesamtfunktion [6, 11], die Denkgeschwindigkeit und das Arbeitsgedächtnis scheint kognitives Training einen steigernden Effekt zu haben. Es gibt allerdings wenig Evidenz dafür, dass die Vorteile kognitiven Trainings über eine Verbesserung in der Trainingsaufgabe hinaus einen positiven Effekt auf die Kognition haben [28] und diese Effekte sind noch weniger verstanden im Kontext der Alzheimer-Erkrankung.

Interventionen zu diesen Risikofaktoren können in Multidomäneninterventionen (Sport, soziale Teilhabe, Ernährung, kognitives Training) kombiniert werden [31, 43].

### Somatische Risikofaktoren

Eine Reihe extrazerebraler Organerkrankungen kann mit kognitiven Defiziten einhergehen und den Verlauf der Alzheimer-Erkrankung beschleunigen. Hier kann es für behandelnde Neurologen und Psychiater von Bedeutung sein, zu verstehen, wie Behandlungen (z. B. Dialyse) die Kognition beeinflussen, bevor z. B. eine krankheitsmodifizierende Therapie der Alzheimer-Erkrankung begonnen wird. Für Spezialisten des betroffenen Organsystems kann es von Bedeutung sein, abzuschätzen, ob sich therapeutische Maßnahmen (z. B. Dialyse) negativ auf die Kognition auswirken. Hier sind also Patienten, Angehörige und behandelnde Fachärzte aller betroffener Disziplinen angesprochen. Notwendig sind hier digitale Lösungen, mit denen kognitive Auswirkungen somatischer Erkrankungen und ihrer Behandlung über verschiedene Disziplinen hinweg erfassbar gemacht werden, und die so die Grundlage dafür bereiten, negative Auswirkungen im Sinne eines präventiven Ansatzes zu minimieren.

Eine abnehmende glomeruläre Filtrationsrate ist mit einem erhöhten Demenzrisiko assoziiert

Zum Beispiel sind Nierenfunktionsstörungen ein potenzieller Risikofaktor für kognitive Störungen und Demenz [4, 26]. Urämische Toxine können direkte neuronale Schäden verursachen. Eine abnehmende glomeruläre Filtrationsrate und Albuminurie sind als Ausdruck einer gestörten Nierenfunktion mit einem erhöhten Demenzrisiko assoziiert. Bei Patienten mit Hämodialyse wird die Inzidenz kognitiver Beeinträchtigungen auf 30–70 % geschätzt [30]. Ein anderes Beispiel ist die hepatische Enzephalopathie (HE), welche ein weites Spektrum subklinischer neurokognitiver Defizite aufweist und von kognitiven Defiziten bis hin zu Desorientierung und Somnolenz reicht [42]. Diabetes und schlechte glykämische Kontrolle sind ein Risikofaktor für HE bei Leberzirrhose [27]. Langjähriger Alkoholkonsum wird mit der Entwicklung von Wernicke-Enzephalopathie, Korsakoff-Syndrom und alkoholbedingter Demenz in Verbindung gebracht [13]. Schließlich können auch alkoholkonsumassoziierte Elektrolytentgleisungen wie eine Hypokaliämie und Hyponatriämie eine HE verstärken [34]. Auch iatrogene Faktoren, wie z. B. der Einsatz von Protonenpumpeninhibitoren [39] oder Opiaten [40], können das Risiko einer HE erhöhen.

### Iatrogene Risiken und Patientensicherheit

Ein wichtiger Aspekt der Prävention der Alzheimer-Erkrankung ist, kognitive Nebenwirkungen medizinischer Maßnahmen zu minimieren. Dies ist in der Versorgungrealität eine besondere Herausforderung, da relevante medizinische Fachdisziplinen oft über den kognitiven Status und das kognitive Risikoprofil der Patienten nicht informiert sind und keine Möglichkeit haben, kognitive Verläufe zu monitorieren. Zudem könnte die fehlende Monitorierbarkeit einer kognitiven Verschlechterung einen negativen Einfluss auf die Berücksichtigung kognitiver Risiken bei der Auswahl der Intervention haben.

Die Verschreibung kognitiv beeinträchtigender Medikamente ist zu vermeiden

Ältere Patienten haben nach einem operativen Eingriff ein erhöhtes Risiko einer POCD oder eines postoperativen Delirs [15]. Ein wichtiger Risikofaktor ist das Vorhandensein einer präoperativen kognitiven Störung, wie einer leichten kognitiven Störung oder einer Demenz [9, 36]. Die Vermeidung der Verschreibung kognitiv beeinträchtigender oder delirfördernder Medikamente anhand der amerikanischen Beers-Kriterien oder der europäischen START/STOPP-Kriterien spielt bei der Prävention kognitiver Störungen eine wichtige Rolle. Chemotherapien können kognitive Störungen („cancer-related cognitive impairment“) induzieren, die durch leichte bis mittelschwere kognitive Defizite gekennzeichnet sind, darunter Beeinträchtigungen der Verarbeitungsgeschwindigkeit, des Gedächtnisses, der exekutiven Funktionen und der Aufmerksamkeit. Bis zu 75 % der Patienten, die mit Chemotherapie gegen Krebserkrankungen außerhalb des Nervensystems behandelt werden, sind von kognitiven Defiziten betroffen [21, 37].

Ein wichtiger Schritt in der Prävention der Alzheimer-Demenz wäre, diese Aspekte der kognitiven Sicherheit in der Versorgung zu berücksichtigen. Das betrifft vor allem Patienten mit einer Alzheimer-Erkrankung und bereits messbaren kognitiven Defiziten (leichte kognitive Störung). Konkrete Empfehlungen hierzu müssen noch in einer interdisziplinären Zusammenarbeit erarbeitet werden.

## Szenarien einer digital unterstützten Prävention in der Versorgung

**Figure 2:**
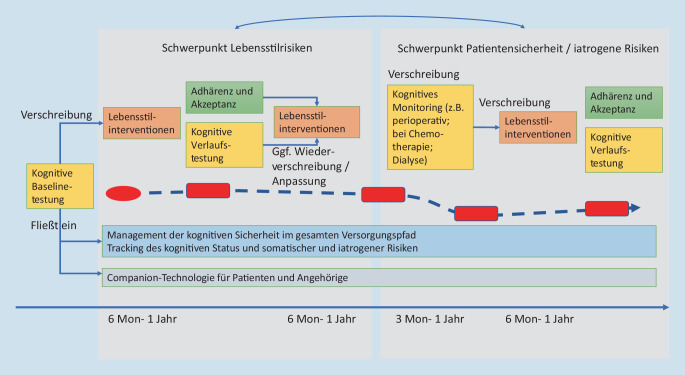

In Abb. 2 ist ein mögliches Szenario dargestellt, wie digitale Technologien zur kognitiven Testung, zur lebensstilrisikobasierten Prävention, zur Prävention somatischer und iatrogener Kognitionsrisiken in die Versorgung eingebunden werden können. Eine wichtige Rolle spielt hierbei die Möglichkeit, die Kognition in der Baseline und im Verlauf bedarfsgerecht und über verschiedene Versorgungskontexte hinweg vergleichbar messen zu können. Diese kontextunabhängige und niederschwellige Messbarkeit ist es, die Prävention motivieren, nachvollziehbar und für Patienten einforderbar machen kann. Ein weiterer zentraler Aspekt ist, dass durch das kognitive Monitoring, die Motivation zur Prävention von der langfristigen Demenzperspektive losgelöst wird und stärker auf die kurzfristige *kognitive Gesundheit *und *kognitive Sicherheit *fokussieren kann.

Das hypothetische Szenario in Abb. 2 zeigt eine Situation, in der einem Patienten nach einer Baseline-Messung eine digital implementierte Lebensstilintervention verschrieben wird. Idealerweise wird hier die Technologie so anpassbar sein, dass der behandelnde Arzt eine individuell bedarfsgerechte Intervention oder eine Kombination von Interventionen und deren Zeitraum (z. B. Sport + MIND-Diät − 12 Monate) auswählen kann. Über die App wird die Adhärenz monitoriert und die kognitive Veränderung nach Ende der Intervention erfasst. Adhärenz und kognitiver Verlauf sind die Entscheidungsgrundlagen für weitere ärztliche Präventionsentscheidungen (Ende der Maßnahme, Verlängerung oder Veränderung der Maßnahme).

Das abgebildete hypothetische Szenario beinhaltet exemplarisch auch eine iatrogene Risikosituation, welche die kognitive Sicherheit des Patienten betrifft: Es ist eine Operation geplant. Hier kann, idealerweise mit der gleichen App, perioperativ untersucht werden, ob die Kognition schlechter wird. In diesem exemplarischen Fall ist eine leichte Abnahme festzustellen. Gemeinsam mit dem Patienten wird daher entschieden, mit einer erneuten Sportintervention zu versuchen, diese Abnahme abzufangen.

## Hürden für die Implementierung

Für die Implementierung eines solchen digital unterstützten Präventionsansatzes existieren eine Reihe von Hürden. Dazu gehören die Akzeptanz digitaler Technologien und die Adhärenz bei der App-Nutzung bei älteren Menschen. Eine aktuelle Studie hat beides untersucht [32] und festgestellt, dass ein höheres Alter stark mit einer geringeren Vertrautheit mit der Smartphone-Technologie und einer weniger häufigen Beschäftigung mit Smartphones und höheren Schwierigkeitsgraden der Smartphone-Nutzung verbunden war. Trotzdem entschied sich die Mehrheit (86,5 %) der älteren Erwachsenen für die Teilnahme an der Smartphone-Studie und zeigte eine außergewöhnlich hohe Adhärenz (85,7 %). Außerdem konnten bei den Teilnehmern weder Vertrautheit mit der Technologie, Technologiewissen, wahrgenommene Schwierigkeit, Geschlecht, Rasse oder Bildung mit der Adhärenz in Verbindung gebracht werden. Diese Ergebnisse deuten darauf hin, dass ältere Erwachsene zwar deutlich weniger vertraut mit der Smartphone-Technologie sind als jüngere Generationen, diese Probleme aber mit einer durchdachten Implementierung, bei der die Unterstützung der Teilnehmer und ein nutzerzentriertes Design im Vordergrund stehen, überwunden werden können.

Die positiven Versorgungseffekte müssen klinisch validiert werden

Eine weitere Hürde ist die Personalisierung und nicht zuletzt die klinische Validierung der positiven Versorgungseffekte, damit diese Angebote erstattet werden können. Für die Personalisierung wird es wichtig sein, dass die verschiedenen kognitiven Testzeitpunkte für das Management der kognitiven Sicherheit im gesamten Versorgungspfad und das Tracking des kognitiven Status und somatischer und iatrogener Risiken digital dokumentiert werden und in der Hand der Patienten jederzeit Behandlern zur Verfügung gestellt werden können. Multidomäneninterventionen zeigen zwar in Teilen positive Effekte auf den Erhalt kognitiver Funktionen [19, 35], werden aber in der Versorgungsrealität personalisierbar werden müssen und in ihrer Zusammensetzung durch die behandelnden Disziplinen an die Bedarfe und Kontraindikationen der Patienten anpassbar sein müssen. Dies ist eine große Herausforderung für die technologische Umsetzung digitaler Lösungen.

Von zentraler Bedeutung wird es sein, durch Companion-Technologien für Patienten und Angehörige Kompetenzen zu stärken. Patienten und Angehörige können durch digitale Companion-Technologien in die Lage versetzt werden, eine Gesundheitsversorgung zu fordern, die ihre kognitiven Beschwerden berücksichtigt, ihre kognitionsbezogenen Gesundheitskompetenzen steigert und ihre kognitive Sicherheit verbessert. Digitale Technologien sind darüber hinaus in besonderem Maße geeignet, patientenrelevante Endpunkte zu erforschen und zu implementieren.

## Fazit für die Praxis

- Das Ziel dieses Konzeptpapiers war es, darzustellen, wie digitale Technologien die Prävention der Alzheimer-Erkrankung verbessern können. Dabei können sie sowohl kognitives Monitoring, kognitive Gesundheit als auch kognitive Sicherheit in der Versorgung verbessern und über verschiedene Punkte des Versorgungspfades hinweg verknüpfen.
- Digitale Technologien können so helfen, das Bewusstsein für die Bedeutung kognitiver Gesundheit und Sicherheit zu verbessern. Die Kopplung von kognitivem Monitoring, kognitiver Gesundheit und kognitiver Sicherheit innerhalb digitaler Technologien kann für Hausärzte und Fachärzte einen Anreiz bieten, kognitive Beeinträchtigungen (und Alzheimer) frühzeitig, im Stadium einer leichten kognitiven Störung, zu diagnostizieren und das interdisziplinäre und gesundheitspolitische Bewusstsein für die Notwendigkeit des Schutzes der Kognition vor der Demenz zu schärfen.
